# Advancing In Vivo Detection of T-Cell Function: Development and Preclinical Evaluation of ^89^Zr-Ivuxolimab, a Human OX40 PET Tracer

**DOI:** 10.2967/jnumed.125.269799

**Published:** 2025-09

**Authors:** Mausam Kalita, Renesmee C. Kuo, Samantha T. Reyes, Deana Rae Crystal Colburg, Irene N. Falk, David Anders, Ophir Vermesh, Samira Hayee, E. Carmen Azevedo, Sydney C. Nagy, Emily M. Deal, Anthony An-Fa Dahm Chen, Christina S. Kong, Federico Simonetta, Anand Giddabasappa, Edmund J. Keliher, Derek W. Bartlett, Kevin P. Maresca, Michelle L. James, Israt S. Alam

**Affiliations:** 1 Molecular Imaging Program at Stanford, Department of Radiology, Stanford University School of Medicine, Stanford, California;; 2 Department of Neurology and Neurological Sciences, Stanford University School of Medicine, Stanford, California;; 3 Department of Pathology, Stanford University School of Medicine, Stanford, California;; 4 Division of Hematology, Department of Oncology, Geneva University Hospitals, Geneva, Switzerland;; 5 Translational Research Center for Oncohematology, Department of Medicine, Faculty of Medicine, University of Geneva, Geneva, Switzerland; and; 6 Worldwide Research, Development and Medicine, Pfizer Inc., New York, New York

**Keywords:** human OX40 receptor, activated T cells, immuno-PET, immunooncology, clinical translation

## Abstract

The variable response to cancer immunotherapies highlights a critical gap in our ability to predict and monitor treatment efficacy. To address this, there is an urgent clinical need for advanced molecular imaging technologies that can noninvasively and precisely assess whole-body immune responses. The OX40 receptor (CD134), a potent costimulatory molecule on T cells, serves as a highly specific marker of T-cell activation, an early and crucial event in immunotherapy efficacy. In this study, we report the development of a human OX40-specific radiotracer based on a clinically evaluated therapeutic—ivuxolimab—and assess its utility for PET imaging of activated T cells in vivo. **Methods:** Deferoxamine conjugation and ^89^Zr radiolabeling were optimized for ivuxolimab. In vitro specificity of the resultant tracer, ^89^Zr-ivuxolimab, was then assessed using primary human T cells and stably transfected human OX40^+ ^(huOX40^+^) human embryonic kidney 293 (HEK293) cells. In vivo specificity and biodistribution of ^89^Zr-ivuxolimab were confirmed in subcutaneously implanted huOX40^+^ HEK293 or parental HEK293 tumor–bearing mice. To evaluate ^89^Zr-ivuxolimab’s utility for detecting T-cell activation in vivo, we used a transgenic human OX40 murine model of acute graft-versus-host disease. Ex vivo gamma counting, autoradiography, and immunohistochemistry were performed to verify tracer-binding specificity. **Results:** ^89^Zr-ivuxolimab was reproducibly synthesized and showed significantly increased in vitro binding to activated human T cells versus resting cells (*P* < 0.0001) and increased binding to huOX40^+^ HEK293 cells versus HEK293 cells (*P* < 0.0001). Longitudinal PET/CT imaging of tumor-bearing mice over 5 d revealed markedly higher tracer accumulation in huOX40^+^ HEK293 tumors compared with HEK293 tumors (*P* < 0.0001). ^89^Zr-ivuxolimab successfully detected T-cell activation in the spleen, mesenteric lymph node, and gastrointestinal tract of mice with graft-versus-host disease induced by transgenic murine T cells expressing human OX40, compared with control groups (total body irradiation, *P* < 0.0001; bone marrow, *P* < 0.001). Ex vivo gamma counting of tissues, autoradiography, and immunohistochemistry corroborated PET findings and confirmed tracer specificity for OX40. **Conclusion:** ^89^Zr-ivuxolimab is a promising radiotracer for clinical translation as an imaging agent for activated T cells. Further investigation of its ability to monitor and predict response to different cancer immunotherapy modalities is warranted.

## Visual Abstract

**Figure:**
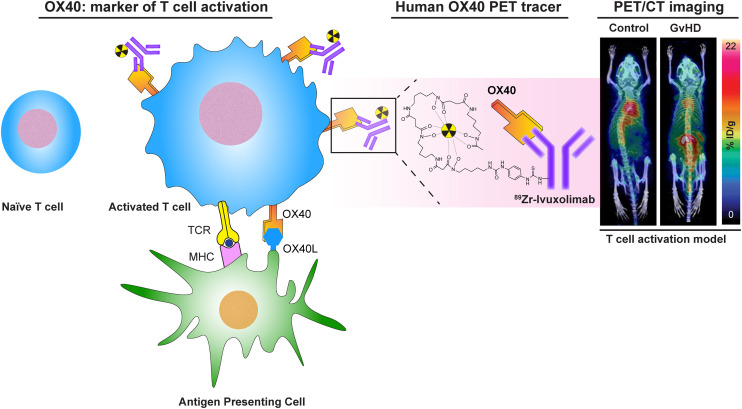

Cancer immunotherapies have revolutionized the treatment of advanced and metastatic disease, offering patients extended survival and, in some instances, complete remission (1,2). However, the variable efficacy observed in patients with cancer, combined with the increased investment in developing and optimizing new treatments, highlights the urgent need for technologies that noninvasively monitor whole-body, longitudinal responses to immunotherapy. Current standard-of-care techniques used to assess immunotherapy efficacy are limited by poor specificity, lack spatial information, or are unable to capture intralesion and interlesion heterogeneity (3,4).

Central to the success of immunotherapy is its ability to prime the patient’s immune system, triggering T-cell activation and subsequent T-cell expansion and tumor infiltration for enhanced killing of cancer cells (5,6). Molecular-imaging technologies, which assess T-cell responses in the tumor microenvironment and beyond, offer a powerful approach to complement standard immune- monitoring practices (7). PET imaging, with its high sensitivity, quantitative capabilities, and clinical availability is ideally suited to provide noninvasive, whole-body insights into T-cell responses (8). Metabolic tracers, such as ^18^F-FDG and others that were later developed for immune imaging (e.g., ^18^F-F-AraG and ^18^F-clofarabine), can exhibit limited specificity for T cells, as targeted metabolic pathways can also be upregulated in other activated immune populations, cancer cells, and adipose tissues (9–13). Thus, CD molecules, which are specific to distinct subsets of immune cells, have emerged as valuable imaging targets. Immuno-PET, which leverages the high specificity of monoclonal antibodies (mAbs) and their fragments, offers a promising approach to visualize these cell surface markers with enhanced precision (14). Thus far, the most clinically advanced T-cell immuno-PET tracers, targeted to cytotoxic CD8^+^ T cells, have shown great potential for delineating anticancer responses (15,16). Although CD4^+^ T cells—crucial for orchestrating a robust T-cell response—have been imaged using PET in preclinical models, this approach has yet to be evaluated in the clinic (17,18). Moreover, imaging constitutively expressed lineage markers, such as CD8 and CD4, does not directly reveal the functional status of cells, such as T-cell dysfunction driven by an immunosuppressive tumor microenvironment. Thus, the imaging of T-cell activation could enable earlier, more accurate prediction of immunotherapy outcomes (19).

We previously identified the OX40 receptor (CD134), a costimulatory molecule on the surface of T cells that can potentiate T-cell receptor signaling, as a promising imaging marker of T-cell activation (20–22). Although absent on resting naïve T cells, OX40 is highly expressed on activated T cells after antigen recognition. Its expression is largely restricted to the T-cell population; this is in stark contrast to other surface activation markers previously evaluated with immuno-PET (e.g., CD25, CD44), which are found on a wide repertoire of immune, endothelial, and cancer cells. The clinical relevance of OX40 in oncology and immunopathology is well documented, providing the rationale for its evaluation as a therapeutic target (23,24). Its prognostic value has also been reported in several cancers, as high OX40 expression in the tumor immune infiltrate taken from patients with cutaneous melanoma, colorectal cancer, or non–small cell lung cancer was associated with improved survival (25–27). We previously demonstrated the broad and predictive utility of murine OX40-specific immuno-PET radiotracers, which enabled early detection of cancer via vaccine-induced T-cell responses (21,28) and early allogeneic immune responses preceding transplant complications in mice (22).

Although there are several ongoing clinical investigations of promising agents (29,30), there are no widely evaluated, gold-standard radiotracers used in the clinical setting to date that can report T-cell activation with high specificity. Given the potential predictive value of OX40 expression, the goal of the current work was to develop a human OX40-specific immuno-PET tracer. Here, we report the development and evaluation of ^89^Zr-ivuxolimab, a PET radiotracer based on ivuxolimab (PF-04518600), a human OX40 receptor agonist. Ivuxolimab, a fully human IgG2 agonist antibody, has demonstrated favorable tolerability at therapeutic doses and confirmed target engagement in phase I and II clinical trials involving patients with locally advanced or metastatic cancers (NCT03217747) (31).

We hypothesize that ^89^Zr-ivuxolimab will serve as a specific radiotracer, enabling comprehensive whole-body assessment of OX40 expression and T-cell activation, making it a promising candidate for future clinical translation for potential early prediction of treatment responses in immunooncology.

## MATERIALS AND METHODS

### Animal Studies

All animal experiments were completed in accordance with the Stanford Administrative Panel on Laboratory Animal Care, which is accredited by the Association for the Assessment and Accreditation of Laboratory Animal Care. Female NOD.Cg-Prkdcscid Il2rgtm1Wjl/SzJ (NSG) (8–10 wk) and female BALB/cJ mice (8–10 wk) were purchased from Jackson Laboratories. Female human *OX40* knock-in transgenic (huOX40tg) C57BL/6 mice (8–10 wk) were purchased from GenOway. Briefly, in this strain, the extracellular part of the murine *OX40* gene is replaced with the human extracellular *OX40* coding sequence, whereas the mouse transmembrane and intracellular parts remain intact (32). All mice were acclimatized for at least 1 wk before the experiments and housed in a temperature-controlled environment under a 12-h light–dark schedule with unrestricted access to food and water.

### Deferoxamine Conjugation and ^89^Zr Radiolabeling

Ivuxolimab, a human OX40-specific antibody (mAb) (PF-04518600; Pfizer Inc.), was conjugated to deferoxamine, using metal-free buffers and our previously described procedures (supplemental materials, available at http://jnm.snmjournals.org) (33). Radiolabeling of deferoxamine–ivuxolimab with ^89^Zr (half-life, 78.4 h) was performed using varying radioactivity-to-conjugate ratios to optimize labeling efficiency and radiochemical purity (supplemental materials).

### huOX40tg Major Histocompatibility Complex–Mismatch Murine Model of Acute Graft-Versus-Host Disease (GvHD)

To assess ^89^Zr-ivuxolimab’s utility for detecting T-cell activation in vivo, we used a modified version of a well-established, major histocompatibility complex–mismatch murine model of acute GvHD (33). Briefly, female BALB/cJ mice received lethal total body irradiation at a total dose of 880 cGy. T-cell–depleted bone marrow (TCD-BM) was obtained from C57BL/6J mice; bones were first homogenized to collect bone marrow suspension, which was subsequently depleted for CD4 and CD8 T cells using MicroBeads (Miltenyi Biotec). Irradiated BALB/cJ mice were injected intravenously with 5 × 10^6^ TCD-BM cells (TCD-BM group). A separate cohort of irradiated BALB/cJ mice also received 1 × 10^6^ splenocytes intravenously, harvested from female huOX40tg C57BL/6 mice to introduce human *OX40* expression. Donor T cells contained within the splenocytes become activated once infused into the BALB/cJ mice, resulting in acute GvHD. Mice were monitored daily, and GvHD scores were assessed on day 5 before PET/CT imaging (34).

### PET/CT Imaging and Image Analysis in Tumor-Bearing Mice and huOX40tg Acute GvHD Model

Mice were anesthetized using isoflurane (2.5%–3.0% for induction and 1.5%–2.0% for maintenance) delivered by 100% oxygen. ^89^Zr-ivuxolimab radiotracer (60 μCi/2.2 MBq; volume, 60 µL of saline; mass dose, 3.2 µg) was administered via the tail vein of mice. PET/CT imaging studies were subsequently performed using the GNEXT PET/CT scanner (SOFIE Biosciences). PET scans (20-min static) were acquired in list-mode format 24 h after injection of ^89^Zr-ivuxolimab and then at 24-h intervals up to 5 d after injection in NSG tumor-bearing mice. Similarly, 20-min static PET scans were acquired 48 h after injection of mice in the huOX40tg acute GvHD study.

The PET system can deliver 0.54 mm of isotropic spatial resolution at the center of the 130-mm field of view. Isotropic resolution was achieved using 3-dimensional ordered-subset expectation maximization reconstruction algorithms with 24 subsets, 3 iterations, and a matrix size of 240 × 240 × 191. All PET scans were followed by a CT scan to provide anatomic reference and PET attenuation correction. Region-of-interest (ROI) analysis of the PET images was performed using a 3-dimensional volume drawing mode around the heart, tumor, thigh muscle, liver, and femur for tumor-bearing mice (Inveon Research Workplace). For the huOX40tg acute GvHD group, ROI analysis of the PET images was performed using a 3-dimensional volume drawing mode around the heart, liver, spleen, thigh muscle, femur, mesenteric lymph node (MLN), and abdomen. PET data were normalized to injected dose and expressed as percentage of injected dose per gram of tissue (%ID/g).

For validation of the PET imaging data, mice were euthanized after their terminal scans, and tissue-associated radioactivity was verified by ex vivo gamma counting (biodistribution analysis), autoradiography, and immunohistochemistry (supplemental materials). Tumor-bearing mice were euthanized at 72 or 120 h after injection of the radiotracer. For the huOX40tg acute GvHD group, terminal scans were acquired at 48 h after injection of the radiotracer.

### Statistical Analysis

Statistical analyses were performed using Prism 9.0 (GraphPad Software). Data were expressed as mean ± SD, with individual values plotted on bar graphs. Statistical significance was determined using unpaired, 2-tailed Student *t* test or 1-way ANOVA with Tukey multiple comparisons test for column and multiple column analyses, respectively. A Bonferroni post hoc test was applied, when appropriate, to correct for multiple comparisons. A *P* value of less than 0.05 was considered statistically significant.

## RESULTS

### Tracer Synthesis

**FIGURE 1.**
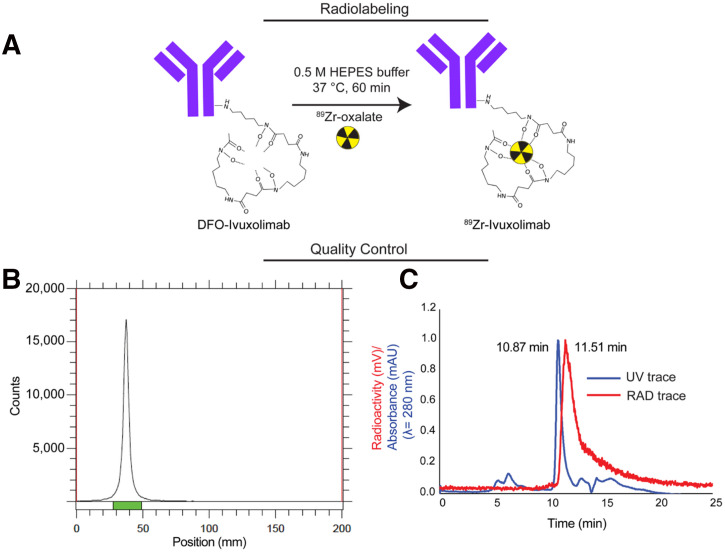
Radiosynthesis of ^89^Zr-ivuxolimab, human OX40 PET tracer. (A) Scheme summarizing optimized conditions for ^89^Zr radiolabeling of deferoxamine (DFO)–ivuxolimab. (B) Radio-iTLC results after labeling and column purification. (C) Radio- and ultraviolet (UV) chromatograms of final tracer product. HEPES = *N*-(2-hydroxyethyl)piperazine-*N*’-(2-ethanesulfonic acid); RAD = radioactivity.

We selected deferoxamine as the chelator for the development of a human OX40 immuno-PET tracer, which is Food and Drug Administration–approved and has a well-established history of success in clinical applications for forming a stable complex with ^89^Zr in humans (15). Deferoxamine conjugation of ivuxolimab was confirmed via electrospray ionization–mass spectrometry, which showed peaks corresponding to unconjugated (*m/z* 146,824.8) and conjugated ivuxolimab with mAb-to-deferoxamine ratios within the desired range of 1:1 (*m/z* 147,576.8) and 1:2 (*m/z* 148,328.5) to preserve immunoreactivity (Supplemental Figs. 1A and 1B). Size exclusion–high-performance liquid chromatography (SEC-HPLC) analysis of the bioconjugate (deferoxamine–ivuxolimab) confirmed a single protein peak (retention time, 10.9 min; Supplemental Fig. 1C). We performed cell-binding studies to ensure that the immunoreactivity of ivuxolimab was preserved after deferoxamine modification (supplemental materials). These studies confirmed that deferoxamine–ivuxolimab binding to human OX40^+^ (huOX40+) human embryonic kidney 293 (HEK293) cells, as detected by a fluorescent antihuman secondary antibody, was comparable to that of the parent mAb, retaining over 94% binding activity (Supplemental Fig. 1D). Subsequent radiolabeling of deferoxamine–ivuxolimab with ^89^Zr-oxalate (Fig. 1A) was highly reproducible, yielding the ^89^Zr-ivuxolimab radiotracer with a mean radiolabeling efficiency (derived from radio–instant thin-layer chromatography (iTLC) of 85.71% ± 2.62% (*n* = 5). After Nap-5 column purification, radio-iTLC confirmed a single peak for ^89^Zr-ivuxolimab (retention factor, 0.2; final radiochemical purity, 98.27% ± 1.73% [*n* = 5]; Fig. 1B), with a specific activity of 18.5 ± 2.91 mCi/mg (684.5 ± 107.3 MBq/mg) (final tracer specifications summarized in Supplemental Table 1). Radio-SEC-HPLC analysis of the final column-purified tracer showed a single peak (retention time, 11.5 min; Fig. 1C), with a corresponding protein peak detected by ultraviolet absorbance (retention time, 10.9 min), confirming the identity of ^89^Zr-ivuxolimab. Radio-iTLC and radio-SEC-HPLC were both routinely used as part of a quality control measure to monitor ^89^Zr-labeled reactions (Supplemental Fig. 2).

### In Vitro Validation of ^89^Zr-Ivuxolimab

**FIGURE 2.**
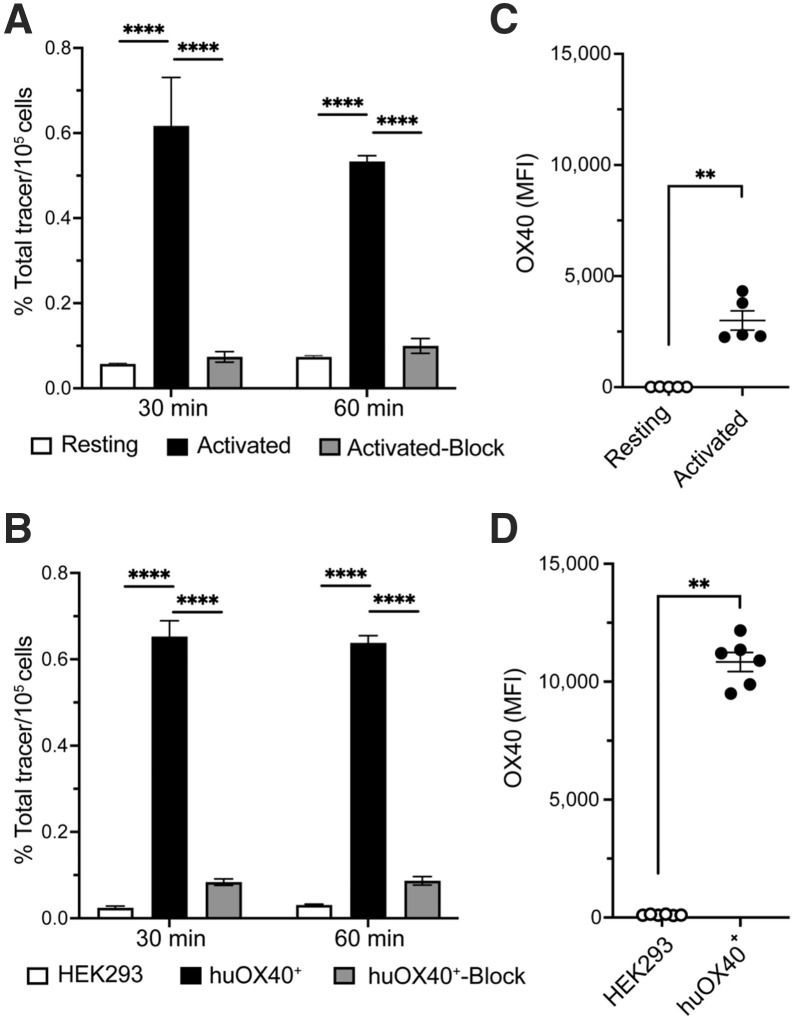
In vitro validation of ^89^Zr-ivuxolimab binding and specificity. (A) ^89^Zr-ivuxolimab binding to resting and activated primary human T cells. (B) ^89^Zr-ivuxolimab binding to parental HEK293 cells and huOX40^+^ HEK293 cells. Binding to both activated T cells and huOX40^+^ HEK293 cells was blocked by coincubation with 25-fold higher mass dose of nonradioactive ivuxolimab. Flow cytometry confirmed increased OX40 expression on activated compared with resting human T cells (C) and huOX40^+^ HEK293 cells (D) compared with parental HEK293 cells. Results pooled from 2 independent experiments. Data presented as mean ± SD (*n* = 5–6 replicates per group). T-cell data are based on cells isolated from 2 healthy donors. **P < 0.01 (1-way ANOVA); ****P < 0.0001 (1-way ANOVA); MFI = mean fluorescent intensity.

In vitro specificity of ^89^Zr-ivuxolimab was evaluated by comparing its binding to isolated primary human T cells that were rested or Dynabead-activated for 48 h (supplemental materials). Significantly higher binding of ^89^Zr-ivuxolimab to activated versus resting human T cells was observed (10.8-fold higher after 30 min of coincubation vs. 7.2-fold higher after 60 min of coincubation, respectively; *P* < 0.0001; Fig. 2A). Tracer binding to recombinant huOX40^+^ HEK293 cells was also significantly higher versus parental HEK293 cells (26.8-fold higher after 30 min versus 20.9-fold higher after 60 min; *P* < 0.0001; Fig. 2B). Coincubation of the radiotracer with a blocking dose of 25-fold higher cold (i.e., nonradioactive) ivuxolimab significantly reduced tracer binding to activated T cells and huOX40^+^ HEK293 cells (by 88% and 87% respectively, *P* < 0.0001), further confirming tracer specificity (Figs. 2A and B). Flow cytometric analysis (supplemental materials) confirmed significantly higher OX40 expression on total activated versus resting human T cells (*P* = 0.0079; Fig. 2C). This increased expression coincided with both CD4^+^ and CD8^+^ T-cell subsets (Supplemental Fig. 3). Initial estimates of surface expression levels (OX40 antigen density) on activated human T cells, yielded between 15,684 ± 50 and 19,469 ± 382 OX40 molecules per activated T cell (Supplemental Fig. 4). Higher target expression on huOX40^+^ HEK293 cells versus parental cells was also confirmed by flow cytometry (*P* = 0.0022; Fig. 2D; Supplemental Fig. 4).

### Detection of Human OX40 In Vivo

**FIGURE 3.**
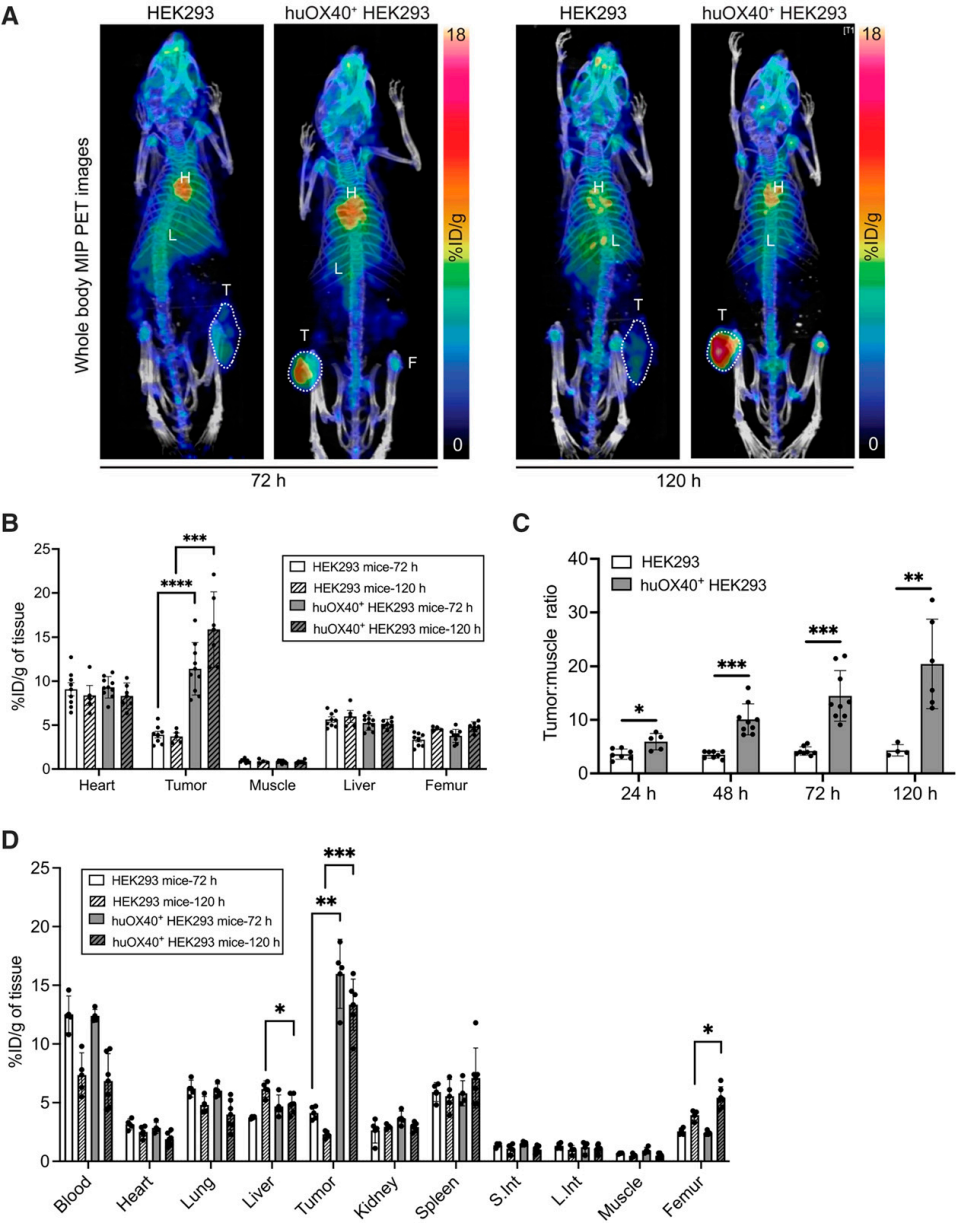
(A) Representative ^89^Zr-ivuxolimab PET/CT whole-body maximum-intensity projection (MIP) coronal images of mice bearing HEK293 or huOX40^+^ HEK293 tumors 72 and 120 h after injection of tracer. White dashed lines indicate tumors (T). (B) Quantification of ^89^Zr-ivuxolimab PET signal 72 (white bars) and 120 h after injection (checked bars) in HEK293 and huOX40^+^ HEK293 tumor–bearing mice. (C) Tumor-to-muscle ratios derived from PET images of huOX40^+^ HEK293 tumor–bearing mice (gray bars) versus HEK293 tumor–bearing mice (white bars). (D) Biodistribution analysis of ^89^Zr-ivuxolimab using ex vivo gamma counting of tissues after necropsy 72 (white bars) and 120 h after injection (checked bars). Data presented as mean ± SD. Results pooled from 2 independent experiments (*n* = 4–9 per group). *P < 0.05; **P < 0.001; ***P < 0.001). F = femur; H = heart; L = liver; L.Int = large intestine; S.Int = small intestine.

Radiometabolism studies in mice confirmed the stability of ^89^Zr-ivuxolimab, supporting its suitability for use as a radiotracer for in vivo imaging applications (supplemental materials; Supplemental Fig. 5). We assessed the in vivo specificity and biodistribution of ^89^Zr-ivuxolimab in a subcutaneous tumor model implanted in NSG mice, using PET/CT imaging. Longitudinal imaging over 5 d after injection showed a markedly higher signal in huOX40^+^ HEK293 tumors versus HEK293 tumors at both 72 and 120 h after injection (Fig. 3A). Quantification of the PET signal, using ROI analysis, confirmed significantly higher tracer accumulation in huOX40^+^ HEK293 tumors compared with the antigen-negative HEK293 tumors (2.9-fold higher at 72 h after injection, *P* < 0.0001; 4.3-fold higher at 120 h after injection, *P* = 0.0007) (Fig. 3B; Supplemental Fig. 6). In both groups of mice, the liver and heart also exhibited a high PET signal, consistent with the expected biodistribution of an intact mAb. Tumor-to-muscle ratios derived from PET quantification were significantly higher for the huOX40^+^ HEK293 tumors versus the HEK293 group at all imaging time points (24 h, *P* = 0.0225; 48 h, *P* = 0.0001; 72 h, *P* = 0.0002; 120 h, *P* = 0.0048) (Fig. 3C). Notably, this tumor-to-muscle ratio increased longitudinally only in the huOX40^+^ HEK293 group (tumor-to-muscle ratios: 24 h, 5.95 ± 0.78; 48 h, 10.1 ± 0.99; 72 h, 14.5 ± 1.60; 120 h, 20.43 ± 3.44) (Fig. 3C).

Biodistribution analysis using ex vivo gamma counting of tissues corroborated PET results, with significantly increased tracer binding in huOX40^+^ HEK293 tumors versus HEK293 tumors 72 h after injection (*P* = 0.0031) and 120 h after injection (*P* < 0.0001) (Fig. 3D; Supplemental Table 2). Autoradiography of tumor and muscle at 120 h after injection enabled high-resolution visualization of radiotracer signal in these tissues (Supplemental Fig. 7A). Quantification of the tissue-associated signal from autoradiography images confirmed higher mean tumor-to-muscle ratios for the huOX40^+^ HEK293 tumor–bearing mice versus the HEK293 tumor–bearing mice (*P* = 0.0005; Supplemental Fig. 7B). Immunohistochemical staining of human OX40 confirmed strong membrane-associated expression in huOX40^+^ HEK293 tumors (Supplemental Fig. 7C).

### Detection of T-Cell Activation In Vivo

**FIGURE 4.**
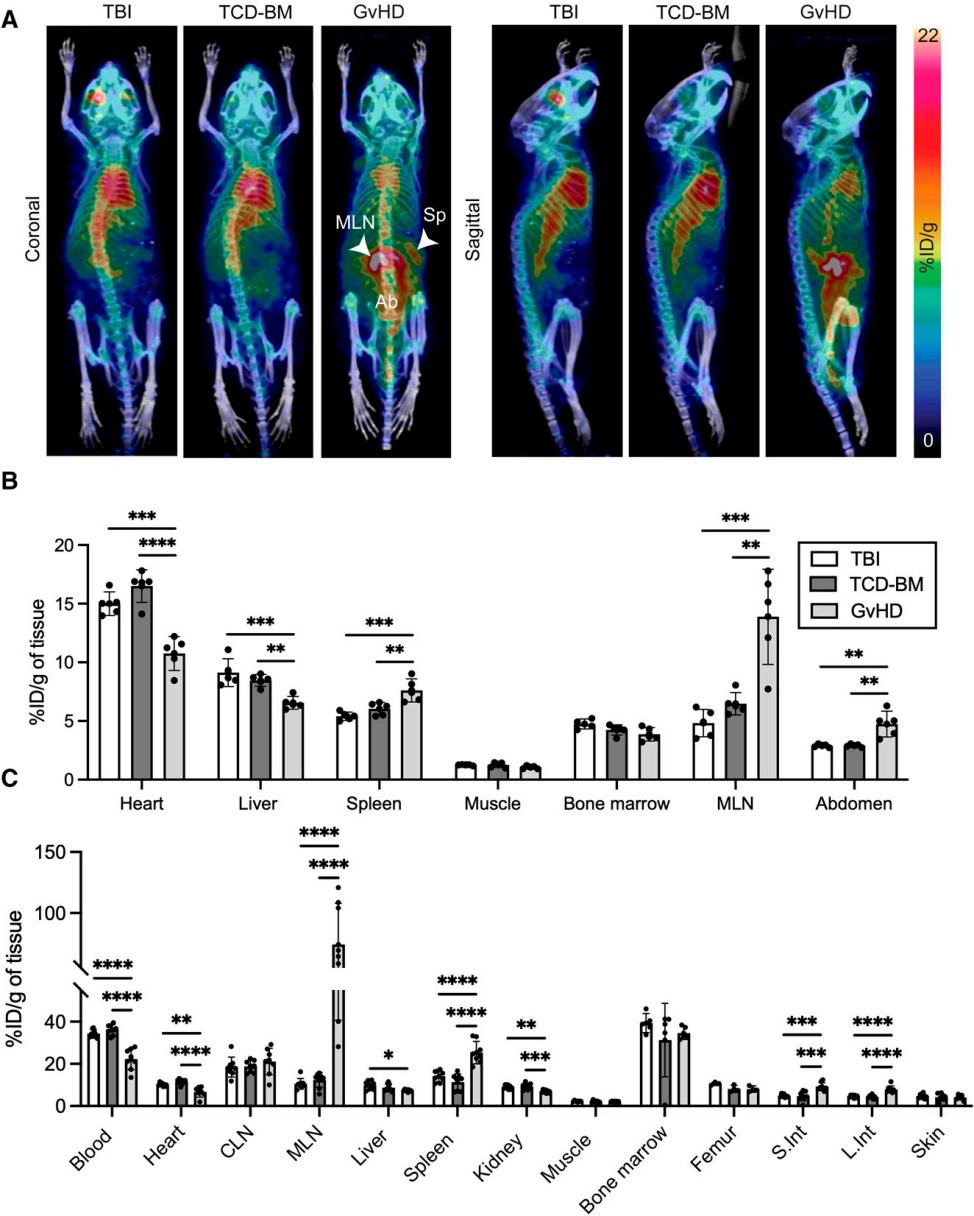
(A) ^89^Zr-ivuxolimab PET allows detection of pathogenic activated T cells in early GvHD. PET/CT whole-body maximum-intensity projection coronal and sagittal images of control total body irradiation (TBI) and TCD-BM mice, shown alongside GvHD mice. (B) Quantification of ^89^Zr-ivuxolimab PET signal 48 h after injection in TBI, TCD-BM, and GvHD groups. (C) Biodistribution analysis of ^89^Zr-ivuxolimab using ex vivo gamma counting of tissues after necroscopy 48 h after injection. Results pooled from 2 independent experiments. Data presented as mean ± SD (*n* = 5–8 mice per group). *P < 0.05; **P < 0.01; ***P < 0.001; ****P < 0.0001. Ab = abdomen; CLN = cervical lymph node; L. Int = large intestine; S.Int = small intestine; Sp = spleen.

**FIGURE 5.**
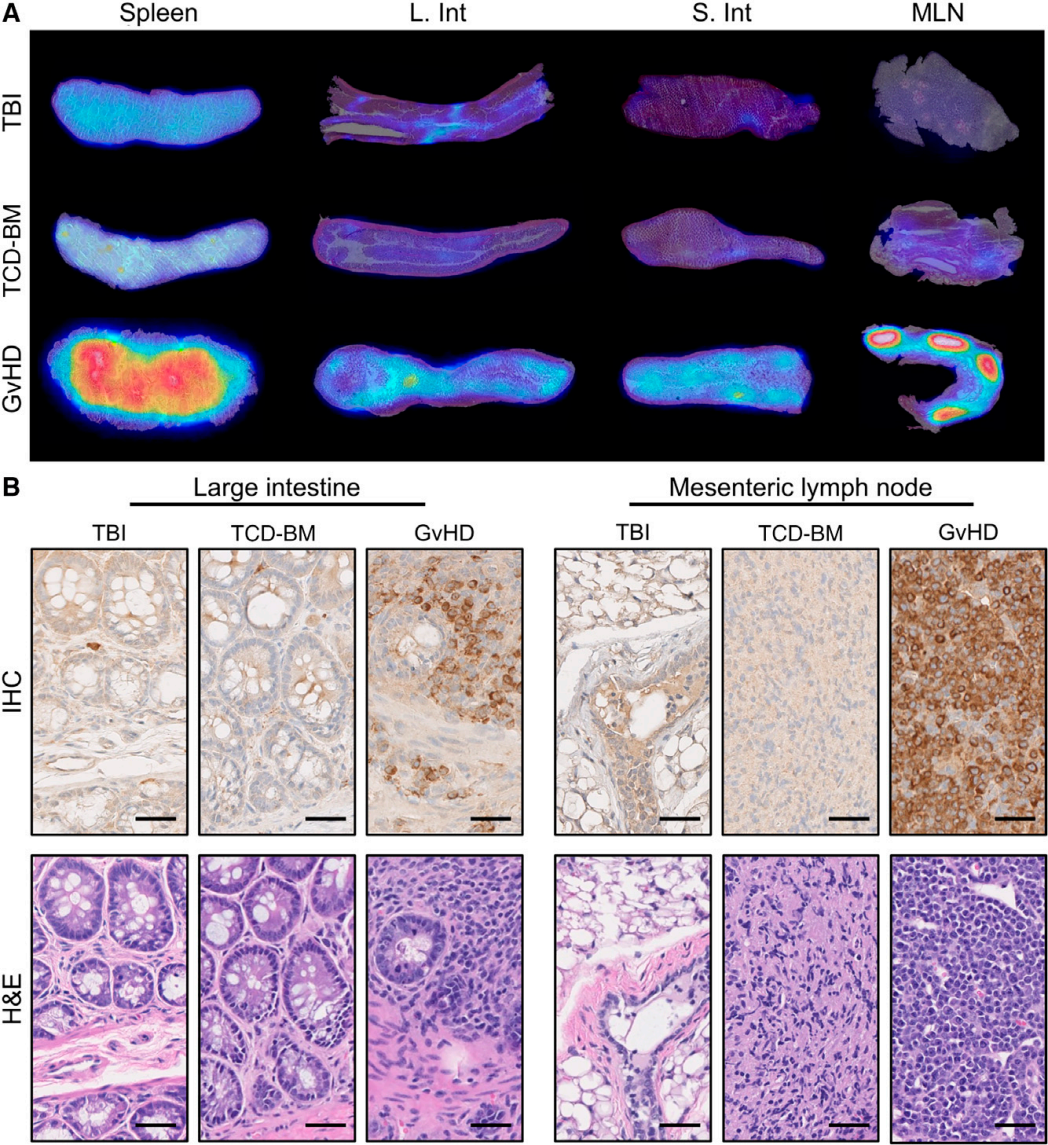
(A) High-resolution visualization of ^89^Zr-ivuxolimab in lymphoid and gastrointestinal tissues via ex vivo autoradiography. (B) Immunohistochemistry (IHC) of tissue sections confirmed presence of huOX40^+^ cells in intestinal tissue and MLN in GvHD mice and lack of huOX40^+^ cells in control total body irradiation (TBI) and TCD-BM mice. Corresponding hematoxylin and eosin (H&E)–stained images of adjacent tissue sections (bottom panel). Histology images were acquired at ×40 magnification. Scale bar = 50 µm. L.Int = large intestine; S.Int = small intestine.

To evaluate the utility of ^89^Zr-ivuxolimab for in vivo detection of activated T cells, we used a modified model of acute GvHD, designed to introduce expression of human OX40 onto murine T cells. Splenocytes were isolated from huOX40tg donor mice and transplanted into recipient BALB/cJ mice. Donor T cells contained within the splenocytes become activated on transfusion, initially proliferating in lymphoid tissues, such as the spleen and MLN of recipient mice, before migrating to GvHD target organs, such as the intestinal tract, to mediate cytolytic attack. On posttransplantation day 5, corresponding with early stages of disease, GvHD mice with minimal overt disease symptoms (mean GvHD score, 0.93 ± 0.70) were injected with ^89^Zr-ivuxolimab, as were control groups (total body irradiation and TCD-BM groups). PET/CT images obtained 48 h after injection (Fig. 4A) and subsequent ROI quantification of images (Fig. 4B) showed a significantly higher PET signal at known sites of T-cell activation and proliferation in the GvHD group, including the spleen (7.61 ± 0.99 %ID/g), MLN (13.89 ± 4.06 %ID/g), and intestines (measured with an abdominal ROI, 4.74 ± 1.1 %ID/g) compared with total body irradiation (spleen, 5.41 ± 0.35 %ID/g, *P* = 0.0007; MLN, 4.82 ± 1.17 %ID/g, *P* = 0.0003; abdomen, 2.90 ± 0.13 %ID/g, *P* = 0.0019) and TCD-BM (spleen, 6.04 ± 0.54 %ID/g, *P* = 0.0086; MLN, 6.48 ± 0.95 %ID/g, *P* = 0.0014; abdomen, 2.90 ± 0.12 %ID/g, *P* = 0.002) control groups. PET findings were further corroborated by ex vivo gamma counting of associated tissues (Fig. 4C; Supplemental Table 3), confirming significantly higher ^89^Zr-ivuxolimab accumulation in the spleen, MLN, and large and small intestines of GvHD mice compared with that in total body irradiation and TCD-BM control mice. Ex vivo macroscopic analysis of ^89^Zr-ivuxolimab using autoradiography enabled high-resolution visualization of tracer distribution in these key tissues (Fig. 5A). A robust autoradiography signal was observed in the intestinal and lymphoid tissues of GvHD mice compared with control groups. Autoradiography of the GvHD spleen clearly visualized spatial overlap of the ^89^Zr-ivuxolimab signal with the splenic white pulp, a region enriched with T cells (Supplemental Fig. 8). An increased ^89^Zr-ivuxolimab autoradiography signal in GvHD lymphoid and intestinal tissues correlated with elevated OX40 expression in these mice, as confirmed by immunohistochemistry (Fig. 5B). These findings reflect the infiltration of pathogenic activated donor T cells in GvHD and further validate the tracer’s specificity.

## DISCUSSION

Monitoring therapeutic responses and predicting clinical outcomes after cancer immunotherapy is challenging due to the complex and variable spatiotemporal immune dynamics. Immuno-PET technologies hold significant promise as integral tools for monitoring and optimizing immunotherapies. Mounting clinical evidence indicates their potential to outperform biopsy measurements of biomarkers (using immunohistochemistry and RNA) by capturing tissue heterogeneity and providing precise, whole-body molecular information to more accurately guide patient management (35,36).

In this study, we report the reproducible synthesis of a first-in-class immuno-PET radiotracer, ^89^Zr-ivuxolimab, to image human OX40, a highly specific marker of T-cell activation. This radiotracer exhibits appropriate in vivo stability, pharmacokinetics, and specificity for human OX40, making it suitable for high-contrast, longitudinal imaging of huOX40^+^ cells in vivo. We provide definitive evidence for tracer specificity using in vitro and in vivo assays using both primary T cells and a huOX40^+^ stable cell line. Importantly, we demonstrated that ^89^Zr-ivuxolimab PET can detect activated T cells in vivo using a GvHD model. In future studies, we plan to evaluate if ^89^Zr-ivuxolimab can be used to monitor the response to clinically relevant GvHD pharmacologic interventions, such as calcineurin inhibitors and cyclophosphamide.

Specific imaging of T-cell functionality is an unmet clinical need that is rapidly gaining momentum. Some promising approaches currently under clinical evaluation include granzyme B (a biomarker of direct killing of target cells mediated by cytotoxic CD8^+^ T cells and natural killer cells) imaging (NCT04169321) (37) and imaging of salvage kinase pathways, which increase in activated T cells (NCT04524195) (29,38). T-cell activation is an early event in the cancer immunity cycle and a critical determinant of treatment efficacy across different cancer immunotherapy modalities. Although emerging tracers targeting other costimulatory receptors (e.g., 4-1BB [CD137]) are providing valuable insights into T-cell activation, further validation in larger and more diverse clinical cohorts is needed to fully assess their potential for guiding immunotherapy protocols and improving outcomes (30). It is crucial for the field to evaluate a variety of T-cell activation markers to identify the most-effective strategies for assessing T-cell function in humans across different immunotherapy modalities, ultimately driving more precise and impactful monitoring of immunotherapy response.

Previously, we demonstrated the utility of murine OX40 immuno-PET for detecting and quantifying early T-cell activation induced by immunotherapy across liquid and solid tumors (20,28). In intratumoral CpG-treated lymphoma tumors, the murine OX40 PET signal correlated with treatment response before the occurrence of gross morphologic changes in the tumor and predicted therapeutic efficacy with greater accuracy than anatomic or blood-based biomarkers alone (20). Additionally, murine OX40 immuno-PET revealed unique insights into the spatiotemporal dynamics of vaccine-induced T-cell responses that occurred beyond the tumor microenvironment, including the requisite immune priming of tumor-draining lymph nodes, which was deemed an identifier of a robust response (20,28). These compelling preclinical findings, coupled with the established clinical relevance of OX40 in immunooncology, were instrumental in driving our efforts to generate a clinically translatable, human OX40 PET radiotracer with the potential to enable early and accurate prediction of immunotherapy response.

Although smaller engineered proteins, peptides, and small molecules continue to be explored for immune imaging, mAbs offer a significant advantage because of their prolonged circulation time (weeks) compared with the shorter half-lives of lower-molecular-weight tracers (hours). This extended circulation provides a longer temporal window for immuno-PET tracers to bind their targets, resulting in higher signal-to-background ratios, the ability to track immune processes longitudinally with a single tracer injection, and enhanced sensitivity, especially for targets low in abundance (as low as ∼10,000 surface molecules per cell) (39). Accordingly, our choice of radiometal, ^89^Zr (half-life, 78.4 h), is well-suited for use with mAbs because it can match their long biologic half-lives. We believe that the described approach is suitable for gleaning first-in-human insights prior to evaluation of smaller mAb fragments, which afford faster pharmacokinetic profiles and are compatible with short-lived isotopes for same-day imaging.

Although our study presents promising results for ^89^Zr-ivuxolimab as a potential clinically translatable imaging agent for T-cell activation, a key limitation is that its limit of detection for OX40 has not been determined. Both receptor density and the number of activated T cells in a given volume are critical factors that will influence successful visualization of these cells in humans. Ongoing antigen density studies and PET imaging in preclinical cancer immunotherapy models using human T cells will help define ^89^Zr-ivuxolimab’s detection threshold for OX40. Other future directions for our work include conducting immunohistochemistry and ex vivo autoradiography to assess OX40 expression in human tumor biopsies, both at baseline and after immunotherapy. These studies will be essential for characterizing changes in OX40 levels over the course of treatment and evaluating its potential to be imaged by ^89^Zr-ivuxolimab.

Generating an OX40 PET tracer based on a clinically evaluated mAb, such as ivuxolimab, allows us to leverage existing pharmacokinetic and safety data, facilitating timely approval of an Investigational New Drug, and advancing its eventual clinical translation. Studies supporting an Investigational New Drug application, which are necessary for the clinical translation of ^89^Zr-ivuxolimab, are ongoing, including radiation dosimetry studies for mouse-to-human estimates and streamlined, reproducible tracer synthesis under current Good Manufacturing Process conditions. Although agonistic engagement of OX40 by ivuxolimab at therapeutic doses has been shown to result in T-cell proliferation, cytokine secretion, and preliminary antitumor activity, we foresee minimal safety concerns in humans because of the low or subpharmacologic mass doses used for imaging. Diab et al. (31) reported agonist effects at 0.1 mg/kg in patients. Although our anticipated mass dose for clinical imaging studies is lower, we will be testing for potential T-cell activation as a precautionary measure. We anticipate that ^89^Zr-ivuxolimab may be instrumental in identifying patients with cancer whose tumors have high basal OX40 expression, pinpointing those patients who are most likely to benefit from OX40 agonist therapy, either alone or in combination with immune-checkpoint inhibitors (27). The tracer may also be invaluable for stratifying responders and nonresponders to immunooncology therapies that increase OX40 expression, enabling timely and precise optimization of treatment protocols (40).

## CONCLUSION

^89^Zr-ivuxolimab is a promising immuno-PET tracer for imaging human T-cell activation. Our data provides definitive evidence of its high specificity for activated primary human T cells and its ability to detect huOX40^+^ activated T cells in vivo. These findings position ^89^Zr-ivuxolimab as a strong candidate for clinical translation, with the potential to guide patient management and improve clinical outcomes by providing early, noninvasive, whole-body monitoring of immunotherapy responses. Future studies are needed to fully assess its safety, sensitivity, and clinical utility for visualizing activated T cells in humans.

## DISCLOSURE

This work was supported by funding from Stanford University’s Department of Radiology gift funds. Israt Alam receives funding from the SNMMI Mitzi & William Blahd, MD, Pilot Research Grant. Israt Alam and Michelle James receive funding from NIH/NCI R01 grant CA286998-02. Anand Giddabasappa, Edmund Keliher, Derek Bartlett, and Kevin Maresca are employees of Pfizer Inc. No other potential conflict of interest relevant to this article was reported.

## Notes

KEY POINTS **QUESTION:** Can a new, human OX40-specific PET radiotracer (^89^Zr-ivuxolimab) be reliably produced, bind to activated human T cells in vitro, and allow precise detection of human OX40 in vivo? **PERTINENT FINDINGS:** ^89^Zr-ivuxolimab can be synthesized reproducibly and demonstrates robust binding to activated human T cells in contrast to resting T cells. This tracer enables longitudinal and highly specific in vivo imaging of human OX40 using PET, as validated in a OX40-expressing tumor model and a T-cell activation model of GvHD. **IMPLICATIONS FOR PATIENT CARE:** Future clinical translation of ^89^Zr-ivuxolimab holds promise for noninvasive and precise interrogation of T-cell activation in patients, potentially enhancing the ability to predict treatment outcomes in cancer immunotherapy, tailoring personalized treatment protocols, and accelerating the development of emerging immunotherapeutic strategies.
